# The Effect of Probiotic *Lactobacillus acidophilus* and Ethanolic Propolis Compound toward Nucleic Acid Deposition in the Extracellular Polymeric Substance of Root Canal Bacteria

**DOI:** 10.1055/s-0042-1750771

**Published:** 2022-07-04

**Authors:** Arya Adiningrat, Rifkhi A. Kusnadi, Asyam S. Allam, Erma Sofiani, Ikhsan Maulana, Hiromichi Yumoto

**Affiliations:** 1 Department of Oral Biology and Biomedical Sciences, School of Dentistry, Faculty of Medicine and Health Sciences, Universitas Muhammadiyah Yogyakarta, Yogyakarta, Indonesia; 2 Molecular Medicine and Therapy Laboratory of Research Division, Faculty of Medicine and Health Sciences, Universitas Muhammadiyah Yogyakarta, Indonesia; 3 Clinical Program of School of Dentistry, Faculty of Medicine and Health Sciences, Universitas Muhammadiyah Yogyakarta, Indonesia; 4 Department of Endodontology, School of Dentistry, Faculty of Medicine and Health Sciences, Universitas Muhammadiyah Yogyakarta, Yogyakarta, Indonesia; 5 Department of Periodontology and Endodontology, Tokushima University Graduate School of Biomedical Sciences, Japan

**Keywords:** ethanolic extract of propolis, extracellular polymeric substance, *Lactobacillus acidophilus*, extracellular nucleic acid, probiotics bacteria, root canal biofilm

## Abstract

**Objective** This study aimed to evaluate the effects of *Apis trigona* ethanolic propolis and probiotic bacteria *Lactobacillus acidophilus* on the nucleic acid concentration in the extracellular polymeric substances (EPS) derived from biofilm of root canal bacteria.

**Materials and Methods** Clinical bacteria of the root canal were cultured with ethanolic extract of propolis (EEP; 10 or 0.1%) and *L. acidophilus* . After the formation of biofilm was observed in the monolayer bacterial culture under several conditions, the enzymatic treatment and nucleic acid quantification were sequentially performed.

**Statistical Analysis** Independent *t* -test and Mann–Whitney were performed following data normality to analyze the significant differences of the treatment effect on the nucleic acid concentration in EPS from the isolated biofilm.

**Results** The results showed that the nucleic acid concentration in EPS biofilm were not increased by coculture with *L. acidophilus* as probiotics. However, the treatment with 10% EEP could significantly increase nucleic acid concentration.

**Conclusion** This study suggested that the biosurfactants from probiotic bacteria *L. acidophilus* might be a promising candidate for endodontic treatment, arguably better than EEP in inhibiting biofilm maturation and complexity.

## Introduction

Persistence of microorganism in the root canal promotes failure in the endodontic treatment. 1 2 This microbial community attaches to the root canal and is able to produce extracellular matrix, forming biofilm to protect the pathogenic community from immune system and external treats such as antibiotic agents. 3 Biofilm complexity and maturation are correspond positively in supporting bacterial infection toward dental tissue, and resulting an inflammation in the targeted area. 4 Therefore, biofilm control is an important factor for the success of endodontic treatment.

Biofilm maturation is also critically attributed by surrounding substances. Among the secreted extracellular substances, extracellular DNA (eDNA) is one of the essential factor of various bacterial pathogenicity which could be produced through bacterial cell autolysin or active secretion. 5 6 It has also been proposed to be a critical factor for the formation and structure complexity of biofilms along with the adhesion of microorganisms. 7 Cell-to-cell interaction and structural integrity of biofilm are strongly affected by the existence of eDNA, since DNase treatment disintegrates biofilm complexity. Furthermore, eDNA also mediates cell adhesion to the host surface through acid base interaction. 6 8 Therefore, by interfering eDNA production and accumulation, it may reduce biofilm pathogenicity.

*Lactobacilli* , a facultative anaerobic lactic acids-producing bacterium, is also known as a major probiotic bacterium having beneficial effect on human health. It also has antagonistic properties against pathogenic bacteria. 9 Many studies revealed that probiotics from *Lactobacilli* family inhibit biofilm formation of pathogenic oral bacteria. 10 11 12 *Lactobacilli* strains produce several antipathogenic organic acid such as lactic acid, acetic acid, and formic acid. 13 They also produce antibacterial polypeptides such as bacteriocin 14 and reuterin. 12 Its inhibition effect can be derived via both coculturing system 13 and secretes incorporation in culture media. 10 11 12 13

In addition to the utilization of antagonist pathogens as a supportive remedy, many researchers have also interested in the biopotential of propolis as a herbal remedy. 15 16 It consists of several active biological organic compounds, such as esters, flavonoids, terpenoids, and phenolic acid, which could also be beneficial for health. Propolis and its derived products have been widely applied in traditional medicine for treating various disease conditions due to their biological and pharmacological properties. 17 Previous research has shown that ethanolic extract of propolis (EEP) could inhibit the proliferation of *E. faecalis* in a concentration-dependent manner. 18 Furthermore, it has also been reported that EEP has antibiofilm activity by reducing biofilm mass analysis. 19 20

Unfortunately, there are some limitations in evaluating root canal biofilm component and also few reports in biofilm analysis using clinical isolated bacteria from the root canal which could be closely related to actual condition in the complex. Therefore, in this study, we would like to observe and focus on the effect of EEP from *Apis trigona* and probiotic bacterium, *Lactobacillus acidophilus,* on the extracellular nucleic acids in biofilm produced by clinical bacteria of the root canal.

## Materials and Methods

### Ethanol Extract of Propolis and Bacteria

As the material of treatment, propolis from *A. trigona* were purchased from the aviary in the Nglipar area, Gunung Kidul, Yogyakarta. It was then extracted by using maceration technique. The propolis were prepared following to previous study with modification. 15 It was initially washed with water, then stirred in 40% ethanol solution for 24 hours. The propolis solution was filtered using filter paper and evaporated through dry heat process (Biobase Biodusty, China). This extraction was performed in the laboratory of Molecular Medicine and Therapy (MMT), Universitas Muhammadiyah Yogyakarta (UMY). Also, 0.1 and 10% EEP concentrations were further applied. *L. acidophilus* (FNCC0051) was purchased from the Food and Nutrition Laboratory of Gadjah Mada University as a probiotic bacterium. While the clinical bacteria sample had been previously isolated and was kindly provided by the MMT Laboratory of UMY.

### Effect of Ethanolic Extract of Propolis on the Growth of Clinical Bacteria from the Root Canal

First, the effect of EEP on the growth of the isolated clinical bacteria was preliminary determined to confirm the similarity in EEP characteristic with our previous study. Clinical bacteria were aerobically cultured in Brain Heart Infusion (BHI: Oxoid, Thermo Fisher, United Kingdom) broth for 24 hours at 37°C, and the optical density of the culture was measured at 600 nm. After 10% EEP treatment, the bacteria were recultured for 24 hours, and the optical density of the bacterial culture was then remeasured. The bacterial culture without 10% EEP was considered as a negative control.

### Measurement of Nucleic Acids Concentration in Extracellular Polymeric Substances

After the preculture of clinical bacteria from a root canal with or without *L* . *acidophilus* FNCC0051 in BHI, EPP was added at a final concentration of 0.1 and 10% in 1.5 mL of bacterial culture and then aerobically cultured in 35-mm dish at 37°C for further 96 hours. Cultured biofilm was rinsed with 1 mL of phosphate-buffered saline (PBS) twice, cultured biofilm was then scraped using a cut tip and suspended with 50 μL of 0.9% NaCl in a 0.2-mL tube. The recovered biofilm was homogenized using an endostraight tip scaler (NSK, Japan) for 1 minute at room temperature, and 10 μL of homogenized bacterial suspension sample transferred into a new 0.2-mL tube was heated for 20 minutes at 96°C. Heated sample mix was then added by 2 μL of Glycobuffer-2 (New England BioLabs B3704S, United Kingdom), 1 μL of Peptide-N-Glycosidase F (PNGase F) (New England BioLabs, United Kingdom), and 7 μL of diethylpyrocarbonate-treated distilled water (DEPC-DW; Himedia TCL016–100ML, India) for the enzymatic treatment for more than 12 hours with 10 seconds for vortexing in the first 4-hour incubation. Overall, 1 μL of proteinase K (QIAGEN 158918, United States) was added after complete enzymatic treatment, then it was further incubated for 30 minutes at 37°C. After the final treatment, the sample was transferred into a 1.5-mL tube containing 479 μL of DEPC-DW and filtered using 0.2 μL filter disk. The same volume of chloroform was then added to the filtrate and vortexed. After the centrifugation at 13,000 rpm for 6 minutes at 25°C, 500 μL of buffer GP2 (Tiangen, China) was added to the aqueous upper phase recovered sample in a new 1.5-mL tube and mixed by inverting. The sample was applied to the CB3 column and centrifuged at 13,000 rpm for 1 minute at 25°C. After discarding the supernatant, 500 μL of GD buffer (Tiangen, China) was added and centrifuged under the same condition. The sample was further suspended into Tris-EDTA (TE) buffer, and the concentration of nucleic acid was measured with Nano-Vue microvolume spectrophotometer (BIOCHROM, United Kingdom).

### Statistical Analysis

The effect of EEP on reducing bacterial growth was indicated as EEP efficacy and was recorded as optical density value of each treatment. The effect of EEP and probiotics coculture on eDNA in biofilm was recorded as nucleic acid concentration (ng/µL). The normality of the data was examined using Shapiro–Wilk test. Student's independent *t* -test and Mann–Whitney *U* -test were used for analyzing the data normally distributed and skewed distributed, respectively. All the statistical analysis was performed using The Statistical Package for the Social Sciences (SPSS) 16.0 software (IBM, Chicago, Illinois, United States) and 5% ( *p* < 0.05) was considered as acceptable significancy level. Data values were stated as value ±  standard of errors (SE).

## Results

### The Effect of Ethanolic Extract of Propolis on the Growth of Clinical Bacteria

**Fig. 1:**
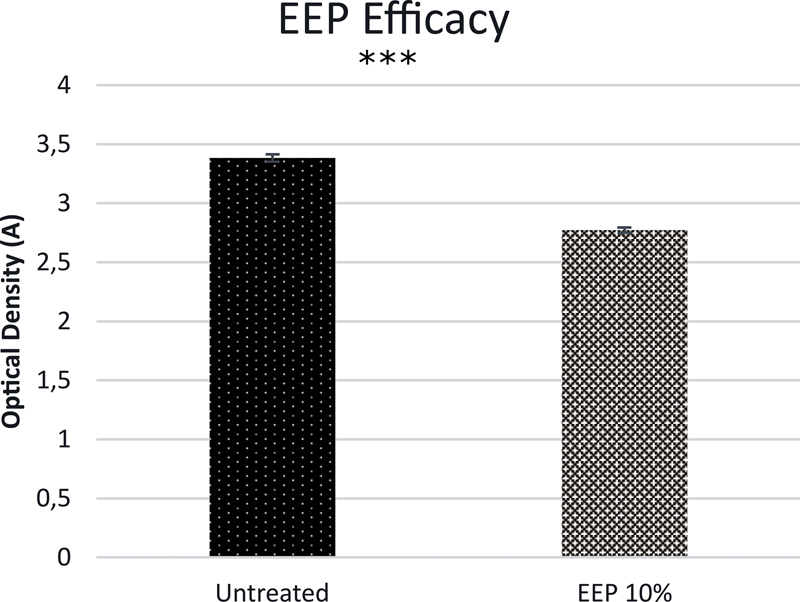
The effect of ethanolic extract of propolis (EEP) on the growth of clinical bacteria from root canal (*** *p* -value < 0.001).

To reconfirm the effect of EEP on the growth of isolated clinical bacteria, the bacteria were cultured in BHI broth with or without 10% EEP for 24 hours. As shown in Fig. 1 , 10% EPP significantly inhibited the bacterial growth (optical density, OD: 2.772 ± 0.0222) compared with untreated control (OD: 3.3827 ± 0.0316), showing that 10% EEP had a bacterial inhibitory effect ( *p <* 0.001; Fig. 1 ).

### The effect of Ethanolic Extract of Propolis on the Concentration of Nucleic Acids in Extracellular Polymeric Substances

**Fig. 2:**
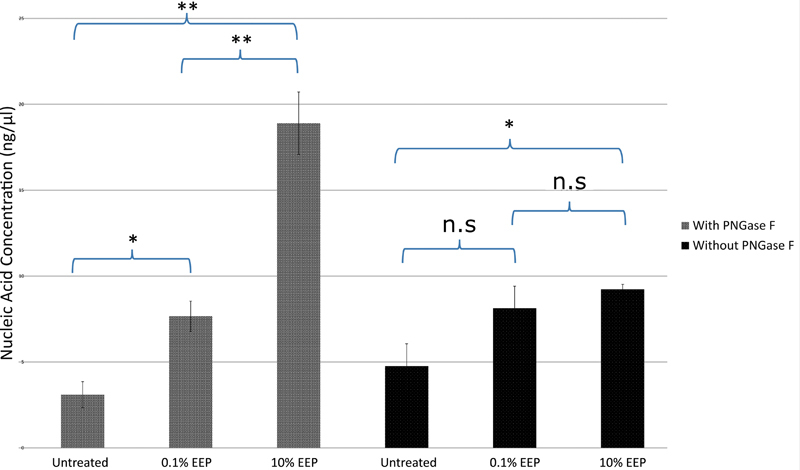
The effect of ethanolic extract of propolis (EEP) on the concentration of nucleic acids in extracellular polymeric substances (EPS) produced from biofilm formed by clinical bacteria from root canal. *p* -Value following Student's independent *t* -test, with PNGase F (untreated: 0.1% EEP = 0.017; untreated: 10% EEP = 0.001; 0.1% EEP: 10% EEP = 0.005). *p* -Value following Student's independent *t* -test, without PNGase F (untreated: 0.1% EEP = 0.139; untreated: 10% EEP = 0.028; 0.1% EPP: 10% EEP = 0.451). With *p* -value ≤ 0.01 is significant (**); *p-* value = 0.01–0.05 is significant (*); *p* -value ≥0.05 is not significant (n.s).

Regarding the extraction of nucleic acids in extracellular polymeric substances (EPS), the effect of enzymatic treatment by PNGase F had also been determined. Further, 0.1% and 10% EEP without PNGase treatment did not show significant difference in increasing nucleic acid concentration ( *p* = 0.451). On the other hand, 10% EEP showed significant difference from 0.1% EEP ( *p* = 0.005) with PNGase F treatment. It significantly increased the concentration of nucleic acids (18.9 ± 1.8193 ng/µL) in EPS ( Fig. 2 ).

### The Effect of Coculture with Probiotic Bacterium, *Lactobacillus acidophilus* , on the Concentration of Nucleic Acids in Extracellular Polymeric Substances

**Fig. 3:**
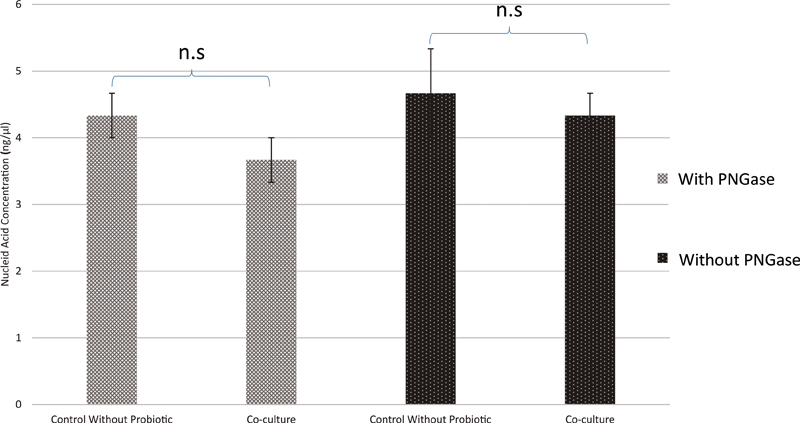
The effect of coculture with probiotic bacterium, *Lactobacillus acidophilus* , on the concentration of nucleic acids in extracellular polymeric substances (EPS) produced from biofilm of root canal clinical bacteria. *p* -Value with PNGase F ( *p-value* = 0.197). *p* -Value without PNGase F ( *p* -value = 0.796). *p* -Value ≥ 0.05 is not significant (n.s).

*L* . *acidophilus* did not affect the concentration of nucleic acids in EPS produced by clinical bacteria from the root canal regardless the PNGase F treatment ( *p* = 0.197 with PNGase F and p = 0.796 without PNGase F; Fig. 3 ).

## Discussion

Bacteria inhabited in the root canal can be attached to the root canal walls and forms biofilm with multiple layers. 4 Biofilm has a complex structure which is influenced by the EPS component. Each component, such as proteins, polysaccharides, and extracellular nucleic acids, affects the biofilm's function and structure. Polysaccharides serve as skeletons in biofilms, and polysaccharide chains in EPS make biofilms resistant of being penetrated by any antibiofilm agent. In addition, nucleic acids in EPS also have a function in improving biofilm stability. 21

Regarding the antibacterial activity of ethanolic propolis, the previous study reported that the ethanolic propolis inhibited the growth of Enterococcus *faecalis* , associated with a significant number of refractory endodontic infections, in a concentration-dependent manner. 18 In this study, the growth of the clinical bacteria was significantly inhibited by 10% EEP, compared with untreated control ( Fig. 1 ). This result showed that the EEP used in this study had antibacterial activity similar to the ethanolic propolis used in previous studies. 18

The nucleic acid in EPS can be accumulated from a variety of sources, such as bacterial. 6 The previous study showed that 0.1% EEP was already toxic to human fibroblasts, suggesting that the toxic effect of 0.1% EEP could also cause bacterial cell lysis. 18 In addition, unpublished data on the previous research showed that the treatment with 10% EEP inhibited the growth of clinical bacteria from the root canal, similar to the result shown in Fig. 1 . However, this inhibitory mechanism by EEP remains unknown, whether it is through the process of bacterial lysis or inhibition of bacterial cells proliferation. 18

Nucleic acid increased by bacterial lysis cannot be easily free from EPS biofilm. Biofilm structure which is composed of proteins and polysaccharides within the EPS complex matrix could be interfered by the release of extracellularly nucleic acids. Mechanical procedures, such as homogenization and vortices, are not enough to remove EPS nucleic acids. However, enzymes can eliminate certain components, including nucleic acids in EPS. For instance, it has been known that three enzymes, that is, *N-* glycanase, dispersin B, and proteinase K, facilitate nucleic acids release from EPS. 22 The function of the enzyme *N* -glycanase is to remove the intact *N-* linked glycinate from glycopeptides and glycoproteins, 23 after the enzyme dispersin B serves to hydrolyze β-substituted *N* -acetylglucosamine. 24 Finally, the enzyme proteinase-K, serine protease with high activity and broad specificity, digests proteins and cleavages peptides. 19 The previous study showed that both *N* -glycanase and proteinase-K had the best results on extracting eDNA from EPS in biofilm samples. 22

Therefore, in this study, the combination of PNGase F, known as *N* -glycosidase F cleaves N-linked (asparagine-linked) oligosaccharides from glycoproteins proteinase K, was used. The concentration of nucleic acids in EPS produced from biofilm formed by clinical bacteria from the root canal was increased under bacterial culture with 10% EEP. The addition of PNGase F in the treatment of 10% EEP increased nearly twice the concentration of nucleic acids released from biofilm EPS compared with that without PNGase F ( Fig. 2 ). The increased effect of EEP under PNGase F treatment in the extraction process of nucleic acids was in a concentration-dependent manner. However, the coculture with a probiotic bacterium, *L* . *acidophilus* , did not affect the concentration of nucleic acids in biofilm formed by clinical bacteria from root canal, regardless of whether it was PNGase F treatment or not ( Fig. 3 ).

Previous research has widely studied the potential of *L. acidophilus* as a probiotic against biofilms. One of these showed that *L* . *acidophilus* could inhibit the formation and growth of biofilms. 25 In addition, *Lactobacillus sp* . as probiotic bacteria has several measures on antibiofilm activity against pathogenic bacteria. 26 One way is to regulate the expression of genes encoding various pathogenic factors, such as glucosyltransferases, which are responsible for strengthening bacterial attachment and increasing the complexity of biofilm. 27 To regulate the targeted genes expression, biosurfactant as an amphiphilic molecule provided by microorganisms, could be essential. 28 Regarding the probiotics' molecular mechanisms to fight the pathogenic bacteria and to manage biofilm, probiotics use several progressive strategies by secreting antagonistic substances against pathogens, inhibiting quorum sensing and biofilm formation, as well as the growth of pathogenic bacteria. 26 On the other hand, substances secreted by probiotics, such as nisin produced by *L. lactis* , are inactive due to their inability to penetrate the bacteria's external membranes, 29 suggesting that some products secreted from probiotics cannot cause bacteriolysis even if at high concentrations. Furthermore, other previous studies using a coculture system reported that biosurfactant from *L* . *acidophilus* could decrease the gene expression of *gtfB* and *gtfC* which played important roles in biofilms and inhibited the growth of bacterial biofilms. 30 In relation to it, this study also showed that the coculture with the probiotic bacterium *L* . *acidophilus* could not increase the concentration of nucleic acids in EPS produced from biofilm formed by clinical bacteria from the root canal ( Fig. 3 ). It further suggested that the probiotic bacteria *L* . *acidophilus* could prevent the maturation and complexity of biofilms. Thus, biosurfactants from *L* . *acidophilus* as probiotic might be a promising candidate for endodontic treatment, arguably better than EEP by inhibiting biofilm maturation.

## Conclusion

Considering several limitations in this study, our finding suggested that probiotics with their biosurfactants could prevent biofilm maturation compared with EEP at high concentration and long duration. Therefore, the current state of our EEP utilization was less recommended due to the possible side effects, regardless of the growth inhibitory effects toward isolated root canal clinical bacteria.
